# Supplemental datasets for examination of the PCAOB's relationship with the audit profession in the United States

**DOI:** 10.1016/j.dib.2020.105743

**Published:** 2020-05-22

**Authors:** Matthew Ege, W. Robert Knechel, Phillip T. Lamoreaux, Eldar Maksymov

**Affiliations:** a Mays Business School, 4353 Texas A&M University, College Station, TX, 77843, USA; b Fisher School of Accounting, University of Florida, Gainesville, FL, USA; c Arizona State University, WP Carey School of Business, School of Accountancy, Arizona State University, PO BOX 873606, Tempe, AZ, 85287-3606 USA

**Keywords:** PCAOB, Audit Regulation, Auditor Inspections, Responsive Regulation Theory

## Abstract

These datasets have been co-submitted to this journal with the research article “A multi-method analysis of the PCAOB's relationship with the audit profession” [1]. The purpose of these datasets is to assist readers of the research article in obtaining further details on two analyses performed therein: a deviant case analysis of the interview data (enclosed here as Dataset 1) and content analysis of disagreement-report pairs (enclosed here as Dataset 2). These datasets could also inform future research into audit regulation by serving as a starting point in developing research ideas or as a context to settings chosen by the researchers for examination. We developed Dataset 1 by examining interviews of eight PCAOB inspectors and six audit partners who were involved in early interactions between the largest firms and the PCAOB for elements of the data that are inconsistent with the explanations and results in the research article. We developed Dataset 2 by identifying audit firm response letters during the period of 2004-2012 where there are one or more instances of explicit disagreement with the PCAOB's inspection results within the response letter. We identified 15 such audit firm response letters and paired them with the subsequent inspection report based on an inspection that started after the audit firm response letter date. We then examined the content of disagreements and the subsequent deficiencies and highlighted issues in these subsequent reports that appear like disagreements.

Specifications table

**Table:** 

**Subject**	Accounting
**Specific subject area**	Regulation of public company auditing in the United States
**Type of data**	Memo Table
**How data were acquired**	Textual analysis of interviews of PCAOB inspectors and audit firm partners. Textual analysis of audit firm response letters and subsequent PCAOB inspection reports.
**Data format**	Raw Analysis
**Parameters for data collection**	For Dataset 1, we compiled data by examining confidential interviews of PCAOB inspectors and audit firm partners. For Dataset 2, we compiled data by examining publicly available audit firm letters and subsequent PCAOB inspection reports.
**Description of data collection**	For Dataset 1, we examined interviews of eight PCAOB inspectors and six audit partners who were involved in early interactions between the largest firms and the PCAOB for elements of the data that are inconsistent with the explanations and results in the research article. We developed Dataset 2 by identifying audit firm response letters during the period of 2004-2012 where there are one or more instances of explicit disagreement with the PCAOB's inspection results within the response letter. We identified 15 such audit firm response letters and paired them with the subsequent inspection report based on an inspection that started after the audit firm response letter date. We then examined the content of disagreements and the subsequent deficiencies and highlighted issues in these subsequent reports that appear similar to prior disagreements.
**Data source location**	Dataset 1: Arizona State University, Tempe, Arizona, United States.
	Dataset 2: The Public Company Accounting Oversight Board, Washington, D.C., United States.
**Data accessibility**	Dataset 1: Access to raw interview data for this study is limited only to the four co-authors of the research article per the requirements of the Institutional Review Board, Arizona State University, United States. Dataset 2: With the article
**Related research article**	Ege, M., W. R. Knechel, P. T. Lamoreaux, and E. Maksymov. A multi-method analysis of the PCAOB's relationship with the audit profession. Accounting, Organizations, and Society (forthcoming).

## Value of the Data

These data provide supplementary materials we mention in our Accounting, Organizations, and Society (AOS) article titled “A multi-method analysis of the PCAOB's relationship with the audit profession”.
Readers of our AOS article, as well as researchers on regulation, could benefit from these data.
These datasets can inform future research on regulation by providing materials to research, as well as insights that may be useful in developing research ideas and performing analysis.

## 1 Data Description

Dataset 1 contains deviant case analysis from interviews of eight PCAOB inspectors and six audit partners in relation to the related research article. The research article uses Responsive Regulation theory [2] as lens in developing empirical hypotheses and interpreting interview-based insights. The deviant case analysis examines the points in our interview data that are inconsistent with the explanations in the research article.

Dataset 2 contains firm-PCAOB disagreement-report pairs during the period of 2004-2012 that we identified in two stages. First, we identified audit firm response letters where there are one or more instances of explicit disagreement with the PCAOB's inspection results within the response letter. We identified 15 such audit firm response letters and paired them with the subsequent inspection report based on an inspection that started after the audit firm response letter date. We then examined the content of disagreements and the subsequent deficiencies and highlighted issues in these subsequent reports that appear similar to disagreements.

## 2 Experimental Design, Materials, and Methods

In compiling Dataset 1, deviant case analysis, we followed the recommendation of Malsch and Salterio [3] on how authors can increase trustworthiness of their qualitative findings (*emphasis* added):

“Trustworthiness of the findings can also increase considerably when the researcher demonstrates they have carried out deviant or negative case analysis. This approach involves searching for, analyzing, and reporting on elements of the data that *do not support, or appear to contradict, patterns of explanations that are emerging from data analysis* (Silverman 2005).” (p. 13)

One of the coauthors performed the deviant case analysis and two other coauthors independently reviewed the data and then the analysis to ensure that the analysis is thorough and includes all deviations.

In compiling Dataset 2, firm-PCAOB disagreement-report pairs during the period of 2004-2012, we performed analysis in two stages. First, we identified audit firm response letters where there are one or more instances of explicit disagreement with the PCAOB's inspection results within the response letter. We identified 15 such audit firm response letters and paired them with the subsequent inspection report based on an inspection that started after the audit firm response letter date. We then examined the content of disagreements and the subsequent deficiencies and highlighted issues in these subsequent reports that appear similar to disagreements. One of the coauthors performed this analysis and two other coauthors independently reviewed the data and then the results of this analysis to ensure that the analysis is thorough, includes all such disagreement-report pairs, and contains accurate descriptions.

## Disclaimer

The views expressed are purely those of the authors and may not in any circumstances be regarded as stating an official position of the PCAOB or any of the audit firms involved.

## Dataset 1: Deviant Case Analysis

### Deviant Case #1 (Anonymous Partner):

Two of the partners believe that there was also ego involved at the PCAOB in response to the firms’ disagreements and that certain lead inspectors took disagreements a lot more personally than others. One of these two partners believes that this was the main driver of the PCAOB's response to the disagreements, while the other partner believes that this was only another factor in addition to risk signaling. Because these are minority views among the partners and the inspectors whom we interviewed, we conclude that this is a deviation that does not materially affect our findings.

### Deviant Case #2 (Anonymous Partner):

In one instance a partner responded to a question about whether the PCAOB used prior inspection reports in their subsequent inspections in the negative. The following was the specific conversation that took place:

Interviewer: ... How did they zero in on areas where they thought, "You know what, we are most likely to find something there." Would they use the prior reports you think?

AP: No, no, no, no. How that worked was ... We know how that works. They all talk ... I don't know if they had a conference call every morning. I'm kidding okay? They talked to all the other teams. They knew exactly where to look.

However, it appears that this response was due to a misunderstanding of the question. As the topic developed further in the interview, it was clear that this partner believed that the PCAOB viewed the firms’ public disagreements as areas of risk to focus on in the subsequent inspections, consistent with our theory. For example, the following subsequent dialogue took place:

Interviewer: …you think they may have been taking some clues from what you [publicly] disagreed about?

AP: Absolutely because let's say you have a professional disagreement over ... Take the [area] issue... We looked at it pretty good… Gave it a look right before we let the reports go out the door. Then you have ... a truly professional disagreement. …What do you do if you truly believe that you have followed the professional guidance and your firm's guidance, which is pretty detailed? It's not like they come in and say, "Well, you didn't follow this part of it." No. You did exactly what the firm told you to do, which was more than what the AICPA auditing standards had to do. You still got written up. If you don't think that'll tick some people off [resulting in a disagreement]? …That becomes one of their ... Okay, they gonna do an inspection, quasi audit. They identify their risk areas. What's their risk area? [A confidential issue X related to an audit standard]. The first thing they want to see [during the next inspection] is “Give me all your [workpapers related to issue X]."

Thus, we conclude that the partner's initial response does not affect our findings because it was due to a misunderstanding of the question and does not reflect this partner's views on whether the PCAOB used public disagreements as signals of risk to focus on during subsequent inspections.

### Deviant Case #3 (Anonymous Partner):

One partner expressed a view about why the firms ultimately stopped disagreeing publicly that was deviant from all other partners. Specifically, we documented the following as part of our summary of the interview with this partner:

This partner mentioned that in the beginning, there was a lot of uncertainty. How will the markets view the reports? Were they going to be a scorecard? After a few years, it became apparent that the market did not care that much about the inspection report. Therefore, there was less of a need to defend the firm's reputation.

In addition, the partner mentioned that there were too many inspection findings mostly related to internal controls around 2010, so it became impractical to make statements about individual deficiencies.

However, in the second part of the interview the partner conveyed that (s)he definitely believes that the PCAOB used disagreements as risk indicators for subsequent inspections. Thus, except for the deviant view of why the firms stopped disagreeing publicly, this partner's views are consistent with our overall findings. Since this partner's view deviates from the rest of our data, we conclude that this view is a minor deviation that does not affect our findings.

### Deviant Case #4 (Anonymous Partner):

One partner noted that from the beginning (s)he accepted the PCAOB as a regulator and did not seem to resent the PCAOB's punitive approach to regulation: “I'd say our firm's view was we can disagree politely. We need to remember they are the regulator.” This partner further said: “We tried to be professional, cordial, respectful and always put in some platitudes around this is good for us and you're doing an important function. And we hope we all improve, getting better going forward. You and us.” Thus, at first, this partner's position seems to contradict our overall findings that the firms resented the PCAOB's punitive approach and resisted its authority.

However, this partner clarified her/his position throughout the interview by explaining that all of the firms misjudged the PCAOB. Moreover, the partner explained that her/his position was somewhat different from the other firms’ leaders in that (s)he saw the need to disagree with the PCAOB and to defend the firm's positions, but wanted to do this more diplomatically than some of the other Big 4 firms:

So early responses [the firms] were first trying to formulate… my thought was we don't need to irritate [the PCAOB] needlessly. But my own personal view… was that we also had to stick up for our partners and our teams that did audits. And if we did not agree then we needed to say so. We shouldn't have [our partners] end up explaining that their audit was deemed an audit failure if all the way up the line [the firm] didn't think it was.

…Again I don't say this to sound altruistic or anything, but it was important. If we want people to grow up wanting to be audit partners and auditors, which is maybe the safest profession one could choose, we had to back them at the same time [by disagreeing with the PCAOB if necessary].

Thus, given the partner's clarification of her/his nuanced position on disagreeing with the PCAOB, this partner's attitude toward the PCAOB appears to be nuanced—the partner did believe that the firms needed to disagree with the PCAOB at times, consistent with our reasoning. Thus, we conclude that overall this partner's view is nuanced, but entirely consistent with our theory.

### Deviant Case #5 (Anonymous Partner):

One partner said the following: “I think a firm's public disagreement to an inspection report was, I won't say completely irrelevant, but couldn't have been a big part of it. There were just too many other things I think that were causing them to say… here's what they think we're doing, or should be doing.” However, in the immediately following sentences the partner clarified “if any one firm was too loud in their response, they probably got a little nod [from the PCAOB] of you know, you're not supposed to be quite that demonstrative [laughs]. … I just don't think that really affected that much, I think it was more all the other things going on that probably drove [the PCAOB inspections], more than that. But again, I would not be telling the truth, there clearly were times that you certainly got the message. We are the regulators, and you are being regulated. And if our teams, our inspection teams, have a judgment about whether you did enough work on the [confidential issue X related to a financial accounting standard], and we think you should have done more, and here's what you should have done, that's the answer, not what you guys think. That at times was the attitude.”

Overall, this partner indicated that (s)he definitely believes that the PCAOB viewed disagreements as risk signals, but that from her/his standpoint there were also major other issues going on that affected the PCAOB's inspections. This partner also acknowledged that some other firms felt that disagreeing with the PCAOB affected them significantly, as this partner explained her/his view on why the firms stopped disagreeing:

I think [the other Big 4 firms] just got the view that it might be construed [by the PCAOB] that the firm doesn't really get it and is not really taking the necessary steps to correct deficiencies if what they're really doing is arguing that they don't agree that there are deficiencies that need to be fixed. And so part of the constructive goal if you want to get better, you're saying you want to get better, we [the PCAOB] are telling you how to get better, and you're telling us we [the PCAOB] don't have a basis for that. I think that's really more of where the dialogue came in. And I'd say there were conversations that one could get the drift. There were probably a few things in writing on just how you process all these reports, but I think the firms got the hint.

Thus, overall, this partner's view is that public disagreements were consequential, but with the nuance that there were other more consequential factors that affected the dynamic between the PCAOB and the firms. This view when taken in its entirety is largely consistent with our theory and does not change our conclusions.

### Deviant Case #6 (Anonymous Inspector):

One inspector had a view that the PCAOB's objective is to identify the right answer for the investors. Initially, this view seems to be more consistent with the persuasion- or cooperation-based approach than with punishment-based approach to regulation:

The biggest thing is getting back to just focusing on the investor, and it is ... Are we getting to the right answer for the investor, and is that investor being protected? That was something you didn't hear at [a Big 4 where this inspector previously worked], at least I didn't. There was a lot around the budget, meeting the budget, collecting the audit fees. So, it was refreshing to get back, and we don't care how long something takes, we just care [about] getting to the right answer… Again, it's all geared towards getting to the right answer, so you're evaluated [as an inspector on] whether or not you're able to get to the right answer. …It's a totally different mindset from the firms, for sure… I remember when I was at the firm, right, it was always PCAOB's the evil company coming in, and be careful, and they're making our lives so difficult. Then I come on the PCAOB and I'm like, yeah, we really don't have an agenda when we go visit an office.

Upon closer analysis, this inspector's view is not inconsistent with our theory because the inspector still believes that it is the PCAOB who has the right answer, not the firms. The inspector also expressed frustration with the firms’ lack of willingness to accept the PCAOB as a regulator who seeks to come up with the right answer. In this inspector's view, the partners lacked this willingness because they did not like being challenged. Overall, though this inspector has a softer take on the PCAOB's mindset, this inspector's views are not inconsistent with our theory. Thus, we conclude that this inspector's softer mindset is a minor deviation that does not affect our conclusions materially.

### Deviant Case #7 (Anonymous Inspector):

One of the inspectors shared his/her opinion that one of the reasons the firm stopped disagreeing with the PCAOB publicly is because they realized that publicly fighting the PCAOB makes them look bad to the public and that the authority-based relationship with the PCAOB, where the firms accept the PCAOB's requirements, actually helps them increase their audit fees. At first, this specific view may seem inconsistent with our theory that the firms stopped because they recognized that the PCAOB will punish them for public disagreements. However, after a deeper consideration of this view, we concluded that this view is still substantively consistent with our theory in that the inspector believes that the firms stopped disagreeing publicly because they realized that they cannot change the PCAOB's conclusions by publicly disagreeing, thereby making themselves look bad to the public. The only inconsistent part is that according to this inspector the firms may have done so to repair reputation and to retain the increased fees. Thus, this is an interesting nuance, but one that is inconsistent with the other inspectors’ views. Therefore, we conclude that this deviant case does not materially affect our conclusions.

### Deviant Case #8 (Anonymous Inspector):

One PCAOB inspector stated toward the beginning of the interview that (s)he does not think that the disagreements affected what the PCAOB inspectors did. However, deeper analysis of this inspector's statement led us to conclude that the inspector merely wanted to state his/her position that the firms’ disagreements do not bias the PCAOB inspectors’ judgment toward a retaliation against the disagreeing firms.

In addition, the inspector immediately clarified his/her position on disagreements by explaining that “if a regulator says this isn't right and then a regulatee says ‘no I disagree with you and I am not going to fix it’ it may have us concentrate even more on this area. This could elevate the concern. Not in retaliation, but more as a normal reaction. If the regulatee says I disagree – this could be a bigger problem we had thought. They are telling us that they are not going to fix it because they think they are doing it correctly. Maybe a planning team thought ‘Geesh they don't think this is a problem, we better focus on this area.’ ”

Thus, though this inspector's initial statement appeared to deviate from our theory, the inspector subsequently elaborated on the statement and clarified his/her position. The clarified position of this inspector is consistent with our theory. Thus, this case does not appear to be inconsistent with our conclusions.

### Overall Conclusion:

We identified each case (or element) within our data where the statement or a series of statements by the interviewees appear to be inconsistent with our theory. We then performed a deeper analysis and concluded that each deviant case is either a minor deviation, a nuance that is consistent with our theory, occurred due to the interviewee's misunderstanding of our question and was later clarified, or a statement that was vague and was later clarified by the interviewee. Overall, we conclude based on our deviant case analysis following the suggestion in Malsch and Salterio [3] that our conclusions reflect our data fairly.

## Dataset 2: Content Analysis of Disagreement-Report Pairs

**Emphasis** is added to indicate issues in the subsequent reports that appear similar to prior disagreements.

**Table:** 

	Audit Firm	PCAOB Inspection Report with the Firm's Disagreement			Subsequent PCAOB Inspection Report with Deficiencies in the Areas Related to Prior Disagreement		
#		Report Date	Audit Firm Response Letter Date	Firm Disagreement Issue in the Accompanying Response	Inspection Start Date	Report Date	Evidence Consistent with the Relationship Between Prior Disagreement and Current Deficiencies or Part II reports
1	Crowe Horwath	June 27, 2008	June 6, 2008	Crowe Horwath made a general statement of disagreement, but areas discussed include extent of testing, hedges, and **allowance for loan losses**.	Aug 1, 2008	May 6, 2009	In this subsequent report, the PCAOB reported a deficiency related to **allowance for loan losses**. By comparison, in its report issued on Sep 24, 2007, prior to the firm's disagreement, the PCAOB did not identify any deficiencies specific to auditing **loans**.
2	Crowe Horwath	May 6, 2009	Apr10, 2009	Crowe Horwath made a general statement of disagreement, but areas discussed include analytic procedures and **allowance for loan losses**.	Aug 1, 2009	June 10, 2010	In this subsequent report, the PCAOB reported a deficiency related to **allowance for loan losses**.
3	Deloitte	Oct 6, 2005	Sep 14, 2005	Deloitte disagreed with deficiencies alleging inappropriate control reliance, failure to consult national office, and insufficient **going concern** considerations.	May 1, 2006	Jun 14, 2007	In this subsequent report, the PCAOB reported a deficiency related to insufficient **going concern** considerations. By comparison, the PCAOB did not report any deficiencies related to going concern in the Deloitte report issued on November 30, 2006, immediately after the disagreement. The fieldwork for the November 30, 2006 report began before the audit firm disagreement expressed in the October 6, 2005 response letter.
4	Deloitte	Nov 30, 2006	Oct 25, 2006	Deloitte disagreed with the deficiencies alleging inadequate evidence related to failure to obtain sufficient competent evidential matter, adequacy of documentation, revenue recognition, passed adjustments, statement of cash flows, **taxes** and **allowance for loan losses**. In addition, for the first time, Deloitte used “strongly disagree” verbiage, compared to “respectfully disagree” in the 2005 response letter.	Mar 1, 2007	May 19, 2008	In this subsequent report, the PCAOB reported several deficiencies related to **loan** work, and one related to **allowance for loan losses** specifically. By comparison, no deficiencies related to **loan** work were disclosed in the report issued on June 14, 2007, immediately after the disagreement. The fieldwork for the June 17, 2007 report began before the audit firm disagreement expressed in the October 25, 2006 response letter. Further, Deloitte was the only firm in this inspection year for which the PCAOB emphasized **tax** work issues in summary comments of its report and made public Part II **tax** work issues. In addition, the PCAOB made an unprecedented statement about Deloitte's overall tone at the top, never repeated in any other report on any firm: “These deficiencies may result, in part, from a Firm culture that allows, or tolerates, audit approaches that do not consistently emphasize the need for an appropriate level of critical analysis and collection of objective evidence, and that rely largely on management representations.”
5	Deloitte	Jun 14, 2007	May 29, 2007	Deloitte disagreed with the deficiencies alleging inadequate work around subsequent activity, promotional allowances, and **deferred tax assets**.	Mar 1, 2008	Apr 16, 2009	In this subsequent report, the PCAOB reported a deficiency related to **deferred tax assets**. The issuer subsequently restated financial statements due to this issue. In this report the PCAOB also made Part II public, alleging inadequate work around **taxes**.
6	Deloitte	May 19, 2008	Apr 30, 2008	Deloitte disagreed with deficiencies alleging departure from GAAP and inadequate work around allowance for loan losses, **income taxes,** and **fair value estimates**.	Oct 1, 2008	May 4, 2010	In this subsequent report, the PCAOB reported a deficiency related to auditing **income taxes** and several deficiencies related to auditing **fair value estimates**. The number of deficiencies related to auditing fair value estimates increased dramatically from one in the April 16, 2009, report to several in this report.
7	Deloitte	Apr 16, 2009	Mar 26, 2009	Deloitte disagreed with deficiencies alleging inadequate work around **ICFR, allowance for loan losses**, and **valuation of securities**.	Oct 1, 2009	Dec 7, 2011	In this subsequent report, the PCAOB reported several deficiencies related to **ICFR, allowance for loan losses,** and **valuation of securities.** By comparison, no deficiencies related to loan work and only one each related to ICFR and valuation of securities were disclosed in the report issued on May 4, 2010, immediately after the disagreement. The fieldwork for the May 4, 2010 report began before the audit firm disagreement expressed in the March 26, 2009 response letter.
8	EY	Jan 11, 2007	Dec 12, 2006	EY made a general disagreement statement, but areas of disagreement included insufficient **analytical procedures**.	Apr 1, 2007	Apr 29, 2008	In this subsequent report, the PCAOB reported a deficiency alleging that the firm used insufficient **analytical procedures**. By comparison, the PCAOB did not report such a deficiency in the report issued on May 2, 2007. The inspection fieldwork for the May 2, 2007 report began before the audit firm disagreement expressed in the December 12, 2006 response letter.
9	EY	May 2, 2007	Apr 5, 2007	EY made a general disagreement statement, but areas of disagreement included long-term licensing agreement and inadequate **documentation** of EY's work, including lacking documentation of work around **fair value estimates**.	Apr 1, 2008	May 19, 2009	In this subsequent report, the PCAOB reported three additional deficiencies alleging inadequate **documentation** of EY's work. The trend in frequencies of these deficiencies is consistent with the proposition that EY's disagreement led to the PCAOB's focus on documentation in EY's subsequent reports. All firms in our sample had **documentation** deficiencies in 2007, but only EY disagreed with these deficiencies. After its disagreement in 2007, it was one of only two firms that did not have a decrease in the number of **documentation** deficiencies in reports issued in 2009 and 2010*.* In addition, both deficiencies for which the client subsequently restated its financial statements related to lacking work around **fair value estimates**.
10	EY	Apr 29, 2008	Apr 24, 2008	EY made a general disagreement statement, but areas of disagreement included internal controls, software development costs, **allowance for loan loss, fair value**, and **underlying data testing**.	Oct 1, 2008	July 2, 2010	In this subsequent report, the PCAOB reported deficiencies related to **allowance for loan loss, fair value**, and **underlying data testing**. Further, the PCAOB issued Part II alleging insufficient evaluation management's estimates and failure to perform **underlying data testing**. By comparison, in the report issued on May 19, 2009, the PCAOB reported only one fair value issue, and no deficiencies related to **allowance for loan loss** and **underlying data testing**. The fieldwork for the May 19, 2009 report began before the audit firm disagreement expressed in the April 24, 2008 response letter.
11	EY	May 19, 2009	May 4, 2009	EY made a general disagreement statement, but areas of disagreement included tax, **fair value** and **loan** auditing.	Mar 1, 2010	Nov 30, 2011	In this subsequent report the PCAOB reported **fair value** audit deficiencies in seven audits with over 30 specific valuation deficiencies in total. The PCAOB also reported several deficiencies related to **loan** testing. In addition, the PCAOB issued Part II alleging insufficient evaluation management's estimates.
12	Grant Thornton (GT)	Nov 30, 2006	Nov10, 2006	GT disagreed with a deficiencies alleging failure to test **controls related to revenue cycle**, using insufficient **analytical procedures**, departure from GAAP (revolving line of credit), and liability recording.	July 1, 2007	Apr 4, 2008	The PCAOB reported deficiencies alleging failure to test **controls related to revenue cycle** and using insufficient **analytical procedures.**
13	Grant Thornton (GT)	Apr 4, 2008	Mar 31, 2008	GT made a general disagreement statement, but did not reference specific deficiencies.	July 1, 2008	July 9, 2009	Since GT did not reference specific deficiencies in its response dated March 31, 2008, we cannot perform content analysis for this case.
14	Grant Thornton (GT)	Jul 9, 2009	June 30, 2009	GT made a general disagreement statement, but did not reference specific deficiencies.	Sep 1, 2009	Aug 12, 2010	Since GT did not reference specific deficiencies in its response dated June 30, 2009, we cannot perform content analysis for this case.
15	PwC	Nov 17, 2005	Oct 26, 2005	PwC made a general disagreement statement, but did not reference specific deficiencies.	May 1, 2006	Oct 18, 2007	Since PwC did not reference specific deficiencies in its response dated October 26, 2005, we cannot perform content analysis for this case.

**Table:** Sources for Inspection Reports Examined for and Referenced in Dataset 2.

Audit Firm	Audit Year	Date of Inspection Report	Link to Inspection Report
BDO	2003	17-11-05	http://pcaobus.org/Inspections/Reports/Documents/2005_BDO_Seidman.pdf
BDO	2004	30-11-06	http://pcaobus.org/Inspections/Reports/Documents/2006_BDO_Seidman.pdf
BDO	2005	16-05-07	http://pcaobus.org/Inspections/Reports/Documents/2007_BDO_Seidman.pdf
BDO	2006	06-05-08	http://pcaobus.org/Inspections/Reports/Documents/2008_BDO_Seidman.pdf
BDO	2007	09-07-09	http://pcaobus.org/Inspections/Reports/Documents/2009_BDO_Seidman.pdf
BDO	2008	12-08-10	http://pcaobus.org/Inspections/Reports/Documents/2010_BDO_Seidman_LLP.pdf
BDO	2009	31-01-12	http://pcaobus.org/Inspections/Reports/Documents/2012_BDO_LLP.pdf
BDO	2010	18-12-12	http://pcaobus.org/Inspections/Reports/Documents/2012_BDO_USA_LLP.pdf
BDO	2011	22-10-13	http://pcaobus.org/Inspections/Reports/Documents/2014_BDO_USA_LLP.pdf
Crowe Horwath	2003	19-01-06	http://pcaobus.org/Inspections/Reports/Documents/2006_Crowe_Chizek.pdf
Crowe Horwath	2004	30-11-06	http://pcaobus.org/Inspections/Reports/Documents/2006_Crowe_Chizek2.pdf
Crowe Horwath	2005	24-09-07	http://pcaobus.org/Inspections/Reports/Documents/2007_Crowe_Chizek.pdf
Crowe Horwath	2006	27-06-08	http://pcaobus.org/Inspections/Reports/Documents/2008_Crowe_Chizek.pdf
Crowe Horwath	2007	06-05-09	http://pcaobus.org/Inspections/Reports/Documents/2009_Crowe_Horwath.pdf
Crowe Horwath	2008	10-06-10	http://pcaobus.org/Inspections/Reports/Documents/2010_Crowe_Horwath_LLP.pdf
Crowe Horwath	2009	28-06-12	http://pcaobus.org/Inspections/Reports/Documents/2012_Crowe_Horwath_LLP.pdf
Crowe Horwath	2010	23-05-13	http://pcaobus.org/Inspections/Reports/Documents/2013_Crowe_Horwath_LLP.pdf
Crowe Horwath	2011	19-12-13	http://pcaobus.org/Inspections/Reports/Documents/2014_Crowe_Horwath_LLP.pdf
Deloitte	2003	06-10-05	http://pcaobus.org/Inspections/Reports/Documents/2005_Deloitte_and_Touche.pdf
Deloitte	2004	30-11-06	http://pcaobus.org/Inspections/Reports/Documents/2006_Deloitte.pdf
Deloitte	2005	14-06-07	http://pcaobus.org/Inspections/Reports/Documents/2007_Deloitte.pdf
Deloitte	2006	19-05-08	http://pcaobus.org/Inspections/Reports/Documents/2008_Deloitte.pdf
Deloitte	2007	16-04-09	http://pcaobus.org/Inspections/Reports/Documents/2009_Deloitte_Touche.pdf
Deloitte	2008	04-05-10	http://pcaobus.org/Inspections/Reports/Documents/2010_Deloitte_Touche_LLP.pdf
Deloitte	2009	07-12-11	http://pcaobus.org/Inspections/Reports/Documents/2011_Deloitte.pdf
Deloitte	2010	28-11-12	http://pcaobus.org/Inspections/Reports/Documents/2012_Deloitte_Touche%20LLP.pdf
Deloitte	2011	07-05-13	http://pcaobus.org/Inspections/Reports/Documents/2013_Deloitte_Touche_LLP_2012.pdf
EY	2003	17-11-05	http://pcaobus.org/Inspections/Reports/Documents/2005_Ernst_and_Young.pdf
EY	2004	11-01-07	http://pcaobus.org/Inspections/Reports/Documents/2007_EY.pdf
EY	2005	02-05-07	http://pcaobus.org/Inspections/Reports/Documents/2007_Ernst_and_Young2006.pdf
EY	2006	29-04-08	http://pcaobus.org/Inspections/Reports/Documents/2008_Ernst_Young.pdf
EY	2007	19-05-09	http://pcaobus.org/Inspections/Reports/Documents/2009_Ernst_Young.pdf
EY	2008	02-07-10	http://pcaobus.org/Inspections/Reports/Documents/2010_Ernst_Young_LLP_US.pdf
EY	2009	30-11-11	http://pcaobus.org/Inspections/Reports/Documents/2011_Ernst_Young_LLP_US.pdf
EY	2010	06-12-12	http://pcaobus.org/Inspections/Reports/Documents/2012_Ernst_Young_LLP.pdf
EY	2011	28-06-13	http://pcaobus.org/Inspections/Reports/Documents/2013_Ernst_Young.pdf
GT	2003	19-01-06	http://pcaobus.org/Inspections/Reports/Documents/2006_Grant_Thornton.pdf
GT	2004	30-11-06	http://pcaobus.org/Inspections/Reports/Documents/2006_Grant.pdf
GT	2005	28-06-07	http://pcaobus.org/Inspections/Reports/Documents/2007_Grant_Thornton.pdf
GT	2006	04-04-08	http://pcaobus.org/Inspections/Reports/Documents/2008_Grant_Thornton.pdf
GT	2007	09-07-09	http://pcaobus.org/Inspections/Reports/Documents/2009_Grant_Thornton.pdf
GT	2008	12-08-10	http://pcaobus.org/Inspections/Reports/Documents/2010_Grant_Thornton_LLP.pdf
GT	2009	29-03-12	http://pcaobus.org/Inspections/Reports/Documents/2012_Grant_Thornton_LLP.pdf
GT	2010	18-12-12	http://pcaobus.org/Inspections/Reports/Documents/2012_Grant_Thornton_LLP_2011.pdf
GT	2011	21-11-13	http://pcaobus.org/Inspections/Reports/Documents/2013_Grant_Thornton_LLP.pdf
KPMG	2003	29-09-05	http://pcaobus.org/Inspections/Reports/Documents/2005_KPMG_LLP.pdf
KPMG	2004	11-01-07	http://pcaobus.org/Inspections/Reports/Documents/2007_KPMG.pdf
KPMG	2005	26-07-07	http://pcaobus.org/Inspections/Reports/Documents/2007_KPMG2006.pdf
KPMG	2006	12-08-08	http://pcaobus.org/Inspections/Reports/Documents/2008_KPMG_LLP.pdf
KPMG	2007	16-06-09	http://pcaobus.org/Inspections/Reports/Documents/2009_KPMG_LLP.pdf
KPMG	2008	05-10-10	http://pcaobus.org/Inspections/Reports/Documents/2010_KPMG_LLP.pdf
KPMG	2009	08-11-11	http://pcaobus.org/Inspections/Reports/Documents/2011_KPMG_LLP_US.pdf
KPMG	2010	15-08-12	http://pcaobus.org/Inspections/Reports/Documents/2012_KPMG.pdf
KPMG	2011	30-07-13	http://pcaobus.org/Inspections/Reports/Documents/2013_KPMG.pdf
McGladrey	2003	30-11-05	http://pcaobus.org/Inspections/Reports/Documents/2005_McGladrey_Pullen.pdf
McGladrey	2004	30-11-06	http://pcaobus.org/Inspections/Reports/Documents/2006_McGladrey_Pullen2.pdf
McGladrey	2005	14-06-07	http://pcaobus.org/Inspections/Reports/Documents/2007_McGladrey_Pullen.pdf
McGladrey	2006	29-04-08	http://pcaobus.org/Inspections/Reports/Documents/2008_McGladrey_Pullen.pdf
McGladrey	2007	06-05-09	http://pcaobus.org/Inspections/Reports/Documents/2009_McGladrey_Pullen.pdf
McGladrey	2008	24-06-10	http://pcaobus.org/Inspections/Reports/Documents/2010_McGladrey_Pullen_LLP.pdf
McGladrey	2009	28-02-12	http://pcaobus.org/Inspections/Reports/Documents/2012_McGladrey_Pullen_LLP.pdf
McGladrey	2010	23-04-13	http://pcaobus.org/Inspections/Reports/Documents/2013_McGladrey_LLP.pdf
McGladrey	2011	27-02-14	http://pcaobus.org/Inspections/Reports/Documents/2014_McGladrey_LLP.pdf
PWC	2003	17-11-05	http://pcaobus.org/Inspections/Reports/Documents/2005_PricewaterhouseCoopers.pdf
PWC	2004	14-12-06	http://pcaobus.org/Inspections/Reports/Documents/2006_PricewaterhouseCoopers.pdf
PWC	2005	18-10-07	http://pcaobus.org/Inspections/Reports/Documents/2007_PricewaterhouseCoopers.pdf
PWC	2006	27-06-08	http://pcaobus.org/Inspections/Reports/Documents/2008_PricewaterhouseCoopers-0627.pdf
PWC	2007	25-03-09	http://pcaobus.org/Inspections/Reports/Documents/2009_PricewaterhouseCoopers-0325.pdf
PWC	2008	12-08-10	http://pcaobus.org/Inspections/Reports/Documents/2010_PricewaterhouseCoopers_LLP.pdf
PWC	2009	08-11-11	http://pcaobus.org/Inspections/Reports/Documents/2011_PricewaterhouseCoopers_LLP.pdf
PWC	2010	27-09-12	http://pcaobus.org/Inspections/Reports/Documents/2012_PricewaterhouseCoopers_LLP-USA.pdf
PWC	2011	20-08-13	http://pcaobus.org/Inspections/Reports/Documents/2013_PwC.pdf

## Declaration of Competing Interest

The authors declare that they have no known competing financial interests or personal relationships which have, or could be perceived to have, influenced the work reported in this article.
